# Rare *Spiroplasma* Bloodstream Infection in Patient after Surgery, China, 2022

**DOI:** 10.3201/eid3001.230858

**Published:** 2024-01

**Authors:** Ningning Xiu, Chao Yang, Xiaowei Chen, Jianping Long, Pinghua Qu

**Affiliations:** Dongguan Kanghua Hospital, Dongguan, China (N. Xiu, J. Long);; Second Clinical Medical College of Guangzhou University of Chinese Medicine, Guangzhou, China (C. Yang, X. Chen, P. Qu);; The Second Affiliated Hospital of Guangzhou University of Chinese Medicine, Guangdong Provincial Hospital of Traditional Chinese Medicine, Guangzhou (P. Qu)

**Keywords:** *Spiroplasma*, bloodstream, infection, surgery, China, bacteria

## Abstract

We report a case of *Spiroplasma* bloodstream infection in a patient in China who developed pulmonary infection, acute respiratory distress syndrome, sepsis, and septic shock after emergency surgery for type A aortic dissection. One organism closely related to *Spiroplasma eriocheiris* was isolated from blood culture and identified by whole-genome sequencing.

*Spiroplasma*, a genus of bacteria in the phylum *Mycoplasmatota*, is characterized by cell structures with no cell walls (*1*). *Spiroplasma* isolates have been primarily reported from plants, guts of insects, tick triturates, and crustaceans (*2*), although a few cases of *Spiroplasma* infection in humans have also been reported, causing cataracts and uveitis in infants and systemic infections in immunocompromised patients (*3*–*6*). We describe a rare *Spiroplasma* bloodstream infection in a patient after surgery for type A aortic dissection in China.

The case-patient, a 68-year-old man, underwent surgery to repair his aorta on June 3, 2022, and he developed a severe respiratory infection afterward while still hospitalized. Fibrobronchoscopy revealed extensive and severe airway erosion, with yellow and thick sputum adhering to the airway walls. A biopsy of a bronchial embolism was taken and sent for examination (Appendix Figure, panel A), and microscopic observation revealed a layered arrangement of thrombi mixed with neutrophils (Appendix Figure, panel B). On June 9, 2022, the patient’s health began to deteriorate (Appendix Table). The patient was diagnosed with pulmonary infection, acute respiratory distress syndrome, sepsis, and septic shock.

Medical staff performed multiple tests on the patient to identify an infectious etiology to explain the patient’s acute illness (Table). *Candida tropicalis* was cultured in bronchoalveolar lavage fluid (BALF) samples. Seven of 12 blood cultures tested positive (Bactec FX; Becton Dickinson, https://www.bd.com/en-us) for a microorganism that was isolated as rare colonies under conditions of 35°C and a 5% CO_2_ atmosphere. Subcultures on Columbia blood agar showed pinpoint-size zones of hemolysis with no macroscopic colony growth at 4 days of incubation; however, Gram stain and Giemsa-Wright stain of the blood could not detect the presence of bacteria. Finally, metagenomic next-generation sequencing was performed on both the blood and BALF samples. Unique reads of *Spiroplasma eriocheiris* (n = 1,577 in BALF, n = 2,344 in blood), human alphaherpesvirus 1 (n = 66,185 in BALF, n = 1,942 in blood), and *Aspergillus fumigatus* (n = 7 in BALF, n = 12 in blood) were detected (Table). We have uploaded raw data to the National Center for Biotechnology Information Sequence Read Archive (BioProject no. PRJNA1021328).

**Table:** Etiologic examination of a postsurgery patient with a blood infection, China, 2022*

Sampling date	Sample classification	Detection technique	Microorganism	Report date
June 11	BALF	Culture	*Candida tropicalis*	June 13
	Hydrothorax	Culture	Negative	June 17
June 12	Blood culture (2 sets)	Culture	Negative	June 18
June 15	Blood culture (2 sets)	Culture	Positive (3 bottles): *Spiroplasma eriocheiris*, identified by 16S rRNA gene sequencing	June 27
	Urine	Culture	Negative	June 18
	BALF	Culture	*Candida tropicalis*	June 17
June 19	BALF	Culture	*Candida tropicalis*	June 22
	Blood culture (2 sets)	Culture	Positive (all): *Spiroplasma eriocheiris,* identified by 16S rRNA sequencing and designated DGKH1	June 27
	Blood	mNGS†	*Spiroplasma eriocheiris* (2,344, 11.36%)	June 20
			Human alphaherpesvirus 1 (1,942, 84.41%)	
			*Aspergillus fumigatus* (12, 0.00%)	
			Human gammaherpesvirus 4 (7, 0.27%)	
			Human betaherpesvirus 5 (3, 0.08%)	
			Human betaherpesvirus 6B (1, 0.04%)	
	BALF	mNGS†	Human alphaherpesvirus 1 (66,185, 99.49%)	June 20
			*Spiroplasma eriocheiris* (1,577, 0.26%)	
			*Candida tropicalis* (42, 0.00%)	
			*Aspergillus fumigatus* (7, 0.00%)	

*BALF, bronchoalveolar lavage fluid; mNGS, metagenomic next-generation sequencing. †Numbers in parentheses indicate unique reads and relative abundance. One set included 2 bottles (1 aerobic and 1 anaerobic).

We characterized the cultivated microorganism, designated DGKH1, by 16S rRNA gene sequencing and whole-genome sequencing analysis. Results of 16S rRNA gene phylogeny show DGKH1 is closely related to *S. eriocheiris* CCTCC M 207170^T^ (Figure). However, the average nucleotide identity value between the genomes of the 2 isolates was 94%, and the average digital DNA–DNA hybridization value between them was 56%, both of which were lower than the threshold values (95%–96% average nucleotide identity and 70% digital DNA–DNA hybridization) used for delineating prokaryotic species (*7*). Therefore, DGKH1 is represented as an unclassified species that is phylogenetically related to *S. eriocheiris*. The 16S rRNA gene sequence (accession no. OQ955597) and genomic DNA sequence (accession no. JASTWG000000000) were deposited into GenBank.

**Figure:**
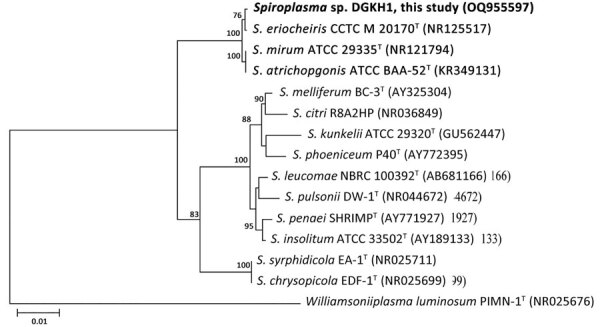
Neighbor-joining phylogenetic tree based on 16S rRNA gene sequences from a postsurgery patient with a blood infection, China, 2022. Tree shows the phylogenetic relationship among the strain DGKH1 from this study (boldface) and closely related species of *Spiroplasma*. *Williamsoniiplasma luminosum* PIMN-1^T^ (GenBank accession no. NR025676) was used as an outgroup in the tree; GenBank accession numbers are provided for all sequences. Bootstrap values (expressed as percentages of 1,000 replications) >70% are shown at the branch points. Superscript T indicates type strains. Scale bar indicates substitutions per nucleotide position.

Results of serum galactomannan testing were negative, and the patient did not respond clinically to voriconazole and caspofungin treatment. We theorize that *C. tropicalis* and *A. fumigatus* played an unlikely role in the patient’s infection, and their detection may reflect colonization or contamination. We postulate that *Spiroplasma* species and human alphaherpesvirus 1 were the main causes of pulmonary infection, acute respiratory distress syndrome, sepsis, and septic shock in this case. Human alphaherpesvirus 1 (previously known as herpes simplex virus 1) is a potential cause of multiorgan failure and septic shock (*8*). Although *Spiroplasma* infection is much less common, the related bacteria *Metamycoplasma hominis* (previously known as *Mycoplasma hominis* and *Mycoplasmoides pneumoniae*) can cause bloodstream infection, pneumonia, and septic shock (*9*). Unfortunately, even with the addition of acyclovir and doxycycline in the therapy, the patient developed multiple organ failure and died on June 23, 2022.

In conclusion, we report a rare case of *Spiroplasma* sp. blood infection in a patient after surgery for type A aortic dissection. *Spiroplasma* is an arthropod-infecting bacterium that may be part of the commensal microbiome of the human gut; there are 13 pieces of relevant information deposited into the gutMEGA database (http://gutmega.omicsbio.info) (*10*). *Spiroplasma* detection is challenging, and the discovery and diagnosis of emerging pathogens, such as the one we have described, can be aided by new technologies such as 16S rRNA gene sequencing and metagenomic next-generation sequencing.

Appendix Additional information for rare *Spiroplasma* bloodstream infection in patient after surgery, China, 2022.
