# Supplementary material for: Rare Spiroplasma Bloodstream Infection in Patient after Surgery, China, 2022
Source: Emerg Infect Dis. 2024 Jan;30(1):187–9. doi: 10.3201/eid3001.230858 (PMC10756377; doi:10.3201/eid3001.230858)
Supplement: Appendix — Additional information for rare Spiroplasma bloodstream infection in patient after surgery, China, 2022. [file 23-0858-Techapp-s1.pdf]

Article DOI: http://doi.org/10.3201/eid3001.230858

*EID cannot ensure accessibility for supplementary materials supplied by authors. Readers who have difficulty accessing supplementary content should contact the authors for assistance.*

# Rare *Spiroplasma* Bloodstream Infection in Patient after Surgery, China, 2022

## Appendix

**Appendix Table**. Blood test results of a postsurgery patient, China, 2022*

| Date | Leukocyte ($10^9$/L) (3.5–9.5) | NEUT% (40%–70%) | PCT (ng/mL) (0–0.046) | CRP (mg/L) (0–10) | IL-6 (pg/mL) (0–7) | Lac (mmol/L) (0.5–2) |
|---|---|---|---|---|---|---|
| June 2nd | 12.13 | 91.2 | NT | NT | NT | 1.1 |
| June 3rd | 18.2 | 86.8 | 23.61 | NT | NT | 1.1 |
| June 4th | 7.67 | 95.0 | 33.29 | 144.72 | NT | 7.4 |
| June 5th | 16.92 | 89.5 | 27.68 | 161.35 | NT | 3.2 |
| June 6th | 14.88 | 93.6 | 14.73 | 89.86 | NT | 1.8 |
| June 7th | 14.61 | 93.8 | 7.25 | 48.7 | NT | 2.9 |
| June 8th | 17.21 | 92.1 | 3.75 | 24.13 | NT | 3.2 |
| June 9th | 21.03 | 87.8 | 2.26 | 34.52 | NT | NT |
| June 10th | 20.77 | 89.9 | 1.34 | >200 | NT | NT |
| June 11th | 22.04 | 89.7 | 1.37 | >200 | NT | NT |
| June 12th | 21.51 | 91.5 | 1.25 | >200 | NT | NT |
| June 13th | 23.28 | 94.8 | 1.25 | >200 | NT | 1.9 |
| June 14th | 23.77 | 96.7 | 1.45 | >200 | NT | 2.2 |
| June 15th | 18.10 | 97.5 | 3.51 | >200 | 1097 | 2.0 |
| June 16th | 19.43 | 98.4 | 4.35 | >200 | 205.1 | 5.4 |
| June 17th | 19.40 | 98.5 | 4.35 | >200 | 232.9 | 6.3 |
| June 18th | 9.08 | 97.0 | 6.74 | 119.53 | 550.6 | 4.0 |
| June 19th | 5.71 | 98.0 | 10.23 | 178.67 | 1558 | 3.2 |
| June 20th | 4.10 | 95.9 | 8.04 | >200 | 975.5 | 3.2 |
| June 21st | 2.66 | 95.9 | 6.15 | >200 | 4120 | 2.9 |
| June 22nd | 9.19 | 77.7 | 8.49 | >200 | 1360 | 7.4 |
| June 23rd | 10.71 | 94.9 | NT | >200 | 4465 | 10.2 |

* CRP, C-reactive protein; IL-6, interleukin-6; Lac, lactic acid; NEUT, Neutrophils; NT: not tested; PCT, Procalcitonin.

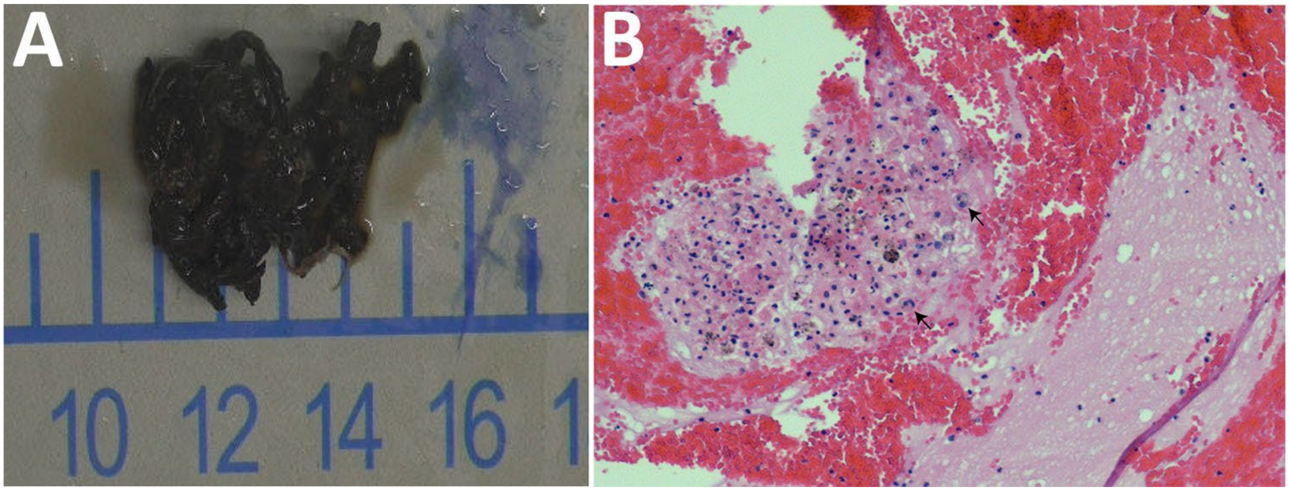


**Appendix Figure**. Images of bronchial embolism from a postsurgery patient, China, 2022. A) Size of the bronchial embolism: 3×3cm. B) Pathological images of bronchial embolism (10×100).
